# IL-37 restrains autoimmune diseases

**DOI:** 10.18632/oncotarget.4887

**Published:** 2015-07-17

**Authors:** Liang Ye, Zhong Huang

**Affiliations:** Institute of Biological Therapy and Shenzhen City Shenzhen University Immunodiagnostic Technology Platform, Shenzhen University, Shenzhen, China

**Keywords:** Immunology and Microbiology Section, Immune response, Immunity

Interleukin-37 (IL-37), a newly discovered member of the IL-1 family, has been identified as a natural inhibitor of immune responses [1]. The human IL-37 gene has been confirmed undergo alternative splicing and exists in five splice variants including IL-37a-e, but a mouse homologue has not been reported [2]. The most glaring is IL-37b that encodes the longest transcript variant includes exons 1 and 2 and expressing an N-terminal prodomain that contains a potential caspase-1 cleavage site, which result in IL-37b precursor cleaved into mature IL-37b [2]. What is noteworthy is that expression of IL-37 is apparently dependent on inflammation milieu and inflammatory cells. We and other studies showed that the expression of IL-37 level is markedly up-regulated in peripheral blood monocular cells (PBMCs), macrophages, epithelial cells, dendritic cells (DCs) and T cells following the stimulation of pro-inflammatory cytokines, e.g. IL-18, IFN-γ, IL-1β and TNF and Toll like receptor (TLR) ligands lipopolysaccharide (LPS), whereas it is lower or not continuously expressed in steady-state target cells and human normal tissues [1–4]. Increasing evidence indicated that modulation of immune system by IL-37 is involved in suppression of innate and adaptive immunity [1–2, 5]. In macrophages and DCs, IL-37 dramatically reduces proinflammatory cytokines secretion, as well as limits them activation and macrophages differentiation. Moreover, IL-37 induced tolerogenic DCs may contribute to induction of regulatory T cells [5]. However, it is still unclear how does IL-37 suppress adaptive immunity by regulating the function of innate immune cells?

In recent years, the powerful inhibitory activity of IL-37 has been investigated in inflammation diseases including LPS induced shock, dextran sulfate sodium (DSS)-induced colitis and obesity-induced insulin resistance [1, 5]. The presence of IL-37 in these inflammation diseases appears to effectively alleviate the excessive inflammatory responses, whereas whether its immunosuppressive features contribute to autoimmune diseases is still poorly investigated. In human studies, our recent results may have clinical implications. Because increased serum IL-37 levels were observed in patients with systemic lupus erythematosus (SLE) [3] and rheumatoid arthritis (RA) [4]. Concomitantly, upregulated IL-37 significantly suppresses the production of proinflammatory cytokines in PBMCs from SLE and RA subjects *in vitro* [3–5]. Tracing the source of IL-37 expression, we clearly observed that CD3^+^ T cells and CD4^+^T cells especially Th1- and Th17 polarization are mainly responsible for the expression of IL-37 in RA [4]. Based on the relationship between IL-37 and adaptive immunity, we therefore asked whether increased IL-37 expression might have a suppressive role in the pathological development of autoimmune diseases.

To address this problem, RA is used as the object of study to reveal the inhibitory effect of IL-37. Despite the etiology of disease still remains unknown, it is clear that a new subset of CD4^+^T cells, the interleukin-17 (IL-17)-producing T helper cells (Th17) cells, are requisite for the pathogenesis of RA, and suppressing IL-17 mediated signal might provide an effective treatments for RA [6]. Strikingly, we found that expression of Th17 related cytokines (IL-17, IL-6 and IL-1β) in CD4^+^T cells from RA subjects were markedly inhibited by recombinant IL-37 while it also decreases the frequency of Th17 cells in RA patients [4], indicating IL-37 can act directly on the adaptive immune response in RA. Considering IL-6 and IL-1β are responsible for inducing the differentiation of Th17, we speculate that IL-37 may affect Th17 cells differentiation and proliferation. Unexpectedly, IL-37 only directly acts on the inhibition of Th17 cells proliferation but not or slightly limits Th17 cells differentiation. Consistently, IL-37 also failed to inhibits the expression of retinoid-related orphan receptor gamma-t (RORγt), a critical Th17 cells transcription factor [4], which further supports this point.

Indeed, the inhibitory effect of IL-37 in pathogenesis of autoimmune diseases also has been confirmed in RA and SLE mice model. We recently revealed that collagen induced arthritis mice treated with adenovirus expressing IL-37 (Ad-IL-37) or recombinant IL-37 exhibited fewer incidences and less clinical and histological arthritis scores, and it appears that the antiarthritic effect of IL-37 was correlated with restraint of IL-17 and Th17 related cytokines [4]. Likewise, our latest findings found that SLE mice injected with Ad-IL-37 attenuates the severity of disease and reduces the pathological character of kidney. Despite IL-37 directly targeted Th17 cells to exert its antiinflammatory role by limiting Th17 cell proliferation, the indirect role in Th17 cells differentiation we still cannot ignore it. Antigen presenting cell (APC) including DCs and macrophages are capable of inducing Th17 differentiation by secreting proinflammatory cytokines such as IL-6, IL-1β and IL-23 [6], whereas the secretions of them were significantly suppressed in APC by IL-37 [1, 5]. While IL-37 receptor IL-1R8 (SIGRR) is abundantly expressed in APC, extracellular IL-37 also as a ligand binds to IL-1R8 result in the inhibition of multiple signal pathways such as MAPKs, NF-KB, TAK1 and Fyn, which exert extracellular anti-inflammatory properties [7]. It would be reasonable to speculate that IL-37 may binds to receptor IL-1R8 on APC to exert its indirectly inhibitory effects on Th17 mediated inflammation response in autoimmune diseases. Considering the fact that unlike conventional cytokines, IL-37 also can be as transcription factor to suppressing inflammation response through a Smad3 dependent mechanism [1], we thus cannot rule out the possibility that intracellular IL-37 mediated immune suppression during the pathogenesis of autoimmune diseases. Given the broadly dual anti-inflammatory properties of IL-37 on inflammatory response, IL-37 might provide an effective therapeutic strategy for autoimmune diseases.

## References

[1] Nold MF (2010). Nat Immunol.

[2] Boraschi D (2011). Eur Cytokine Netw.

[3] Ye L (2014). J Transl Med.

[4] Ye L (2015). J Immunol.

[5] Chen HM (2015). Cytokine.

[6] Martinez GJ (2008). Ann N Y Acad Sci.

[7] Nold-Petry CA (2015). Nat Immunol.

